# Evaluation of diagnostic and predictive values of the serum VEGF-A level and systemic immune-inflammation index in small cell lung cancer

**DOI:** 10.7150/jca.51972

**Published:** 2021-01-01

**Authors:** Jing-hao Yao, Yu Shao, Jun-jun Wang, Yu-long Li, Han-qi Yang, Jing Liu, Yan Yang

**Affiliations:** 1 Department of Medical Oncology, The First Affiliated Hospital of Bengbu Medical College, Bengbu, Anhui, People's Republic of China.; 2 Department of Radiation Oncology, The First Affiliated Hospital of Bengbu Medical College, Bengbu, Anhui, People's Republic of China.; 3 Department of Surgical Oncology, The First Affiliated Hospital of Bengbu Medical College, Bengbu, Anhui, People's Republic of China.; 4 Department of Clinical Laboratory, The First Affiliated Hospital of Bengbu Medical College, Bengbu, Anhui, People's Republic of China.

**Keywords:** small cell lung cancer, vascular endothelial growth factor, systemic immune‐inflammation index, diagnosis, prediction, prognosis

## Abstract

**Purpose:** To evaluate diagnostic and predictive values of the serum vascular endothelial growth factor-A (VEGF-A) level and systemic immune-inflammation index (SII) in small cell lung cancer (SCLC) patients.

**Methods:** From January 2018 to April 2020, we prospectively enrolled 59 untreated SCLC patients in the study group and 50 non-neoplastic patients in the control group. Blood samples were collected at baseline, after the first two cycles of chemotherapy and at progression in the study group and at entry in the control group. Serum VEGF-A was measured by chemiluminescence, SII was calculated based on complete blood count results, and the relationship between the VEGF-A/SII and clinicopathological characteristics, chemotherapeutic efficacy and progression-free survival (PFS) of SCLC patients was analyzed.

**Results:** Baseline serum VEGF-A was significantly higher in SCLC patients than in non-neoplastic patients (*P<*0.001), while baseline SII was not (*P=*0.114). There was no correlation between baseline VEGF-A and SII in SCLC patients (*P=*0.123); however, there was a significant correlation between baseline VEGF-A and disease stage and central nervous system (CNS) metastasis (*P=*0.021 and *P=*0.012, respectively), as well as between baseline SII and disease stage and liver metastasis (*P=*0.026 and *P=*0.018, respectively). Serum VEGF-A was significantly lower than the pretreatment level after 2 cycles of treatment (*P=*0.049) but was not different at progression (*P=*0.247). Baseline VEGF-A was correlated with the treatment response of first-line chemotherapy (*P=*0.001), while baseline SII was not (*P=*0.392). Kaplan-Meier survival analysis suggested that the PFS of first-line chemotherapy was significantly longer in the low-VEGF-A group at baseline than the high-VEGF-A group (11.37 vs. 6.17 months, *P<*0.001). There was a trend toward longer PFS of first-line chemotherapy in the low-SII group at baseline than the high-SII group, but the difference was not significant (12.10 vs. 9.10 months, *P>*0.050). Univariate and multivariate Cox regression analyses suggested that baseline VEGF-A (HR: 3.443, 95% CI: 1.330-8.908, *P=*0.011) was an independent prognostic factor for PFS in SCLC patients.

**Conclusions:** Baseline serum VEGF-A and SII are associated with important clinicopathological characteristics of SCLC patients. VEGF-A, but not SII, has the ability of diagnosis and predicting first-line chemotherapeutic efficacy and prognosis in SCLC patients.

## Introduction

Lung cancer is the most prevalent malignancy worldwide, with small cell lung cancer (SCLC), the most malignant subtype of lung cancer, accounting for approximately 10-15% of all lung cancer patients 1, 2. Presently, SCLC remains a difficult disease to treat; mostly diagnosed at an advanced stage, with rapid progression, and prone to early metastasis and resistance to conventional chemotherapy and radiotherapy. Patients with limited-stage SCLC generally survive less than 2 years, while those with extensive stage SCLC survive less than 1 year 3. Therefore, it is necessary to discover new efficacy and prognostic indicators to predict clinical outcomes and optimize treatment options for these patients.

Angiogenesis is an important biological prerequisite for tumor proliferation and metastasis 4. Vascular endothelial growth factor (VEGF) is considered an important regulator of angiogenesis. Activation of the VEGF pathway promotes cell proliferation, migration and endothelial cell invasion and mediates tumor angiogenesis 5. Within the VEGF family, VEGF-A plays a key role in tumor-associated angiogenesis 6, and elevated VEGF-A is associated with a poorer prognosis in a variety of malignancies 7, 8. The immune-inflammatory status of the tumor microenvironment (TME) also plays an important role in the development of malignant tumors, promoting tumor cell proliferation and survival, angiogenesis and tumor metastasis and decreasing tumor sensitivity to antineoplastic drugs 9, 10. In recent years, as a novel index, the systemic immune-inflammation index (SII) has come to be considered as a better indicator of systemic immune-inflammatory status compared to traditional indexes, such as the neutrophil-lymphocyte ratio or platelet-lymphocyte ratio, and some studies have found an adverse association between the SII and the prognosis of various tumors, including lung cancer 11, 12.

There remains a lack of convenient and effective predictive markers for SCLC, and the relationship between angiogenesis and the immune-inflammatory response is unknown. Previous clinical studies evaluating the relationship between serum VEGF and the SII and SCLC are limited and mainly involve the prediction of prognosis, some of which are controversial 13-17, and few studies have evaluated the predictive value of the treatment efficacy of VEGF and the SII. Therefore, it is necessary to perform a comprehensive evaluation of the diagnostic, therapeutic efficacy and prognostic value of VEGF and the SII in patients with SCLC. In the present study, we propose to perform a combined assay for serum VEGF-A and the SII to evaluate their predictive value on the efficacy and prognosis of patients with SCLC.

## Methods

### Patient selection

From January 2018 to April 2020, we prospectively enrolled 59 patients who attended the First Affiliated Hospital of Bengbu Medical College for the primary treatment of SCLC. The inclusion criteria were as follows: patients with histologically confirmed SCLC; patients who had not received antitumor therapy against SCLC; patients who were not suitable for or were unwilling to undergo surgery or radiation therapy; and patients with target lesions that could be evaluated for efficacy. The exclusion criteria were as follows: a combination of other tumors or subtypes; patients with severe cardiac, hepatic, or renal disease; and patients with severe bleeding or infectious diseases. There were 45 males (76.3%) and 14 females (23.7%) age 45 to 78 years, with an average age of 63.6 years, enrolled in the study group; there were 24 smokers (40.7%), and the median Eastern Cooperative Oncology Group performance status (ECOG PS) score was 0 (0-2) in the study group. Tumor staging was based on clinical, pathological, and imaging data using TNM staging (AJCC 8th edition). There were 35 patients with stage II-III, 24 patients with stage IV, 4 patients with central nervous system (CNS) metastasis, and 8 patients with liver metastasis. In addition, 50 non-neoplastic patients without bleeding or infectious diseases were enrolled as controls during the same period, including 38 (76.0%) men and 12 (24.0%) women age 39-79 years, with an average age of 62.8 years, and 20 (40.0%) smokers. The two groups were comparable in terms of baseline information such as sex, age, and smoking history.

### Therapeutic regimen and follow-up

All patients received at least one cycle of combination chemotherapy containing etoposide and platinum (etoposide 80-100 mg/m^2^ days 1-3 combined with cisplatin 25 mg/m^2^ days 1-3 or carboplatin AUC 5-6 day 1, every 3 weeks as one cycle) as the first-line therapy according to the NCCN guidelines 18, with a maximum of six cycles of chemotherapy. Patients' clinicopathological data were collected through case data and followed up by telephone or hospital review until progression or loss to follow-up, with a median follow-up time of 9.1 months (1.5-24.2 months). Progression free survival (PFS) was calculated from the start of chemotherapy until disease progression, death, or loss to follow-up.

### VEGF-A detection and SII calculation

We collected 5 ml of peripheral venous blood from the patient before the first cycle of treatment (baseline), before the second and third cycles of treatment, and at the time of progression detection, centrifuged at 3000 rpm for 10 minutes, and quickly stored the supernatant in a -20 °C refrigerator for reserve. As is standard, VEGF-A detection was performed using a Weigao JR-1 Chemiluminescent Immunoassay Analyzer and Vascular Endothelial Growth Factor Assay Kit (chemiluminescence) (Shandong Weigao Group Medical Polymer Co., Ltd., Weihai, China) according to the manufacturer's instructions, and the technicians did not have access to the clinical data. A complete blood count was performed using a Sysmex XE-2100 fully automatic hematology analyzer with original matching reagents, calibration and quality control products (Sysmex Corporation, Kobe, Japan), and the SII was calculated using the following formula: SII = platelet count ×neutrophil count/lymphocyte count.

### Chemotherapeutic efficacy assessment

The chemotherapeutic efficacy was evaluated according to the Response Evaluation Criteria in Solid Tumors (RECIST) version 1.1 every 2 cycles 19, and the patient's optimal therapeutic effect was recorded as described here. Complete response (CR): disappearance of all target lesions; partial response (PR): ≥30% reduction in the sum of the baseline lesion longitudinal diameter; stable disease (SD): reduction in the sum of the baseline lesion longitudinal diameter without reaching PR or increase in the sum of the baseline lesion longitudinal diameter without reaching PD; progressive disease (PD): a ≥20% increase in the sum of the long diameters of the baseline lesions or the appearance of new lesions; CR and PR as effective treatment, SD and PD as ineffective treatment.

### Statistical analysis

Statistical analysis was performed using SPSS 24.0 for Windows (SPSS Inc., Chicago, IL, USA). According to a t-test, the age data in this study obeyed a normal distribution and were described by means. Other continuous data, such as VEGF-A and SII, did not obey a normal distribution and were described as medians (lower quartile-upper quartile). The sensitivity and specificity of VEGF-A and SII were determined by receiver operating characteristic (ROC) curves, and the areas under the curves (AUC) were calculated. Continuous variables of two groups of independent samples were compared using the Mann-Whitney U test, and continuous variables of two groups with correlated samples were compared using the Wilcoxon signed rank test. Continuous variables of multiple groups with correlated samples were compared using the Friedman test, and when there were statistically significant differences between the groups, further two-by-two comparisons were made using the Friedman test and corrected using the Bonferroni method. Correlations between the two variables were analyzed using Spearman's correlation test. A survival analysis was performed using the Kaplan-Meier method, and comparisons of survival between the two groups were performed using the log rank test. Prognostic factors were initially screened using a univariate Cox regression analysis, with test levels set at α = 0.05; factors with *P>*0.05 were included in the multivariate Cox regression analysis, and hazard ratios (HR) and corresponding 95% confidence intervals (CIs) were calculated. All tests were two-sided and the test level was α=0.05.

## Results

### Diagnostic values of baseline serum VEGF-A and SII in SCLC

**Figure 1:**
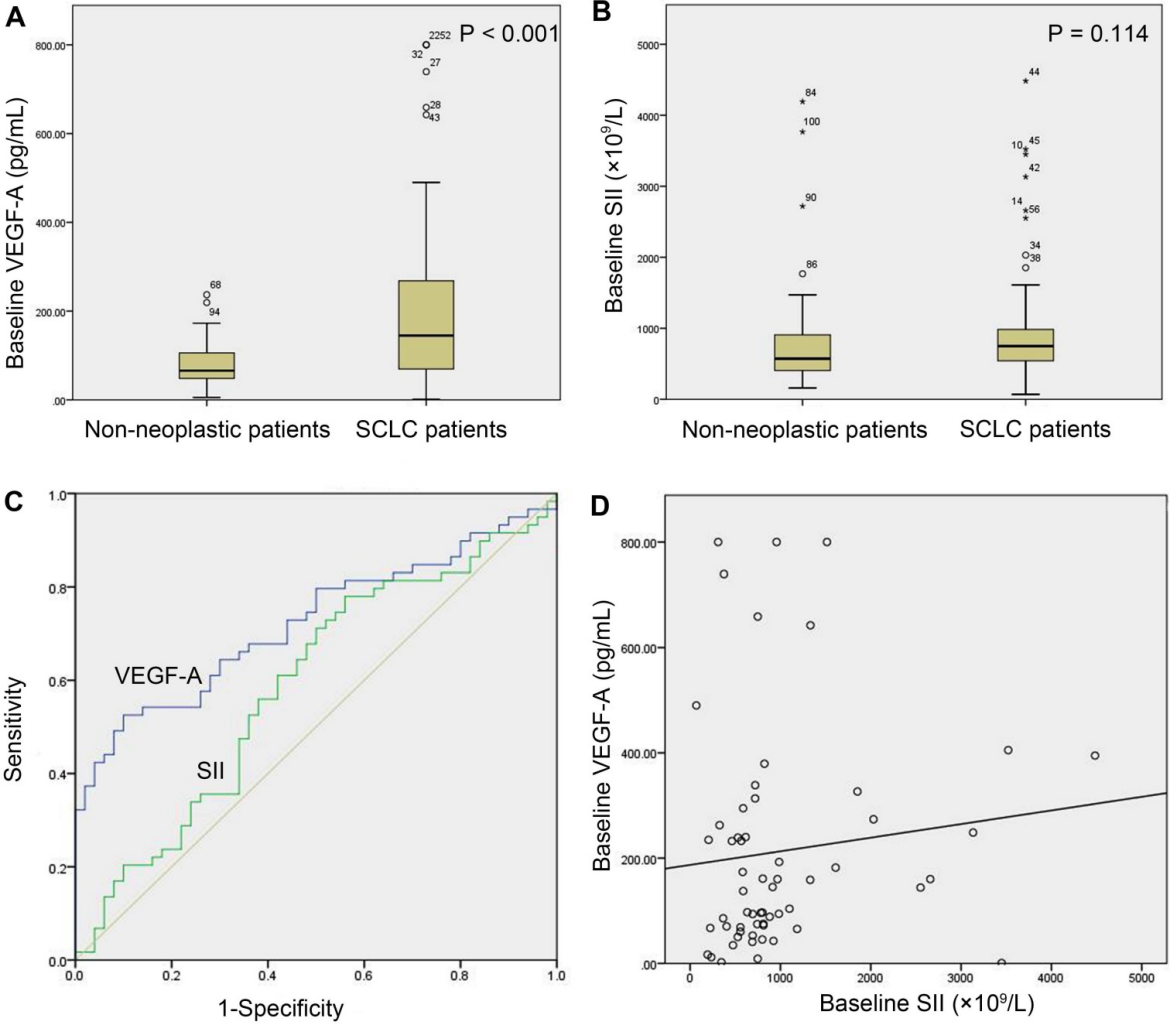
** Baseline serum VEGF-A and SII in SCLC versus non-neoplastic patients. (A)** Baseline VEGF-A levels were significantly higher in SCLC patients (n=59) than in non-neoplastic patients (n=50) (P<0.001); **(B)** SCLC patients (n=59) and non-neoplastic patients (n=50) did not show a significant difference in baseline SII (*P*=0.114); **(C)** The AUC of the ROC curve of baseline VEGF-A was 0.723 (95% CI: 0.629-0.804, *P*<0.001). The baseline SII did not show diagnostic value in SCLC (*P*=0.113); **(D)** There was no significant correlation between baseline VEGF-A levels and the baseline SII in SCLC patients (n=59, *r*=0.203, *P*=0.123). Note: In (A) and (B), numbers represent the sample number, and circles and asterisks indicate outliers and extremes in the sample data, respectively. Abbreviations: CI: confidence interval; SCLC: small cell lung cancer; SII: systemic immune-inflammation index; VEGF-A: vascular endothelial growth factor A.

The baseline serum VEGF-A levels were significantly higher in the study group (n=59) than in the control group (n=50) (145.10 (68.74-273.84) vs. 65.89 (48.19-106.24) pg/mL, *P<*0.001, Figure 1A). The baseline SII of the study group was not significantly different from that of the control group (748.30 (528.57-985.83) (×10^9^/L) vs. 571.84 (401.73-922.15) (×10^9^/L), *P=*0.114, Figure 1B). The AUC of the ROC curve of baseline VEGF-A was 0.723 (95% CI: 0.629-0.804, *P*<0.001). With a cut-off value of 140.92 pg/ml, VEGF-A had a sensitivity of 52.54%, a specificity of 90.00%, a positive likelihood ratio (LR+) of 5.25, and a negative likelihood ratio (LR-) of 0.53 (Figure 1C). The SII did not show diagnostic value in SCLC (*P*=0.113) (Figure 1C). There was no significant linear correlation between baseline VEGF-A and the SII in the study group (*r*=0.203, *P=*0.123, Figure 1D).

### Relationships between baseline serum VEGF-A/SII and clinicopathological characteristics of SCLC patients

The relationships between baseline VEGF-A/SII and the clinicopathological characteristics of SCLC patients are shown in Table 1. Baseline VEGF-A was significantly associated with disease stage (*P=*0.021) and CNS metastasis (*P=*0.012), and baseline SII was significantly associated with disease stage (*P=*0.026) and liver metastasis (*P=*0.018). There were no significant correlations with sex, age, smoking status, or ECOG PS (all *P>*0.05).

### Dynamic changes of serum VEGF-A before and after treatment in SCLC patients

In the study group, there were 46 patients with VEGF-A data from more than 2 cycles of treatment, and the dynamics of serum VEGF-A in these patients during treatment are shown in Table 2. The serum VEGF-A level was 148.06 (58.46-299.45) pg/mL before treatment and 90.59 (42.92-218.58) and 90.14 (49.94-132.61) pg/mL after the first and second cycles of treatment (*P*=0.047), respectively, which showed a significant reduction after 2 cycles of treatment compared to pretreatment (*P=*0.049). A total of 29 patients had complete VEGF-A data for each cycle and at progression, and the dynamics of serum VEGF-A in these patients before treatment and at progression are shown in Table 3. The VEGF-A level of pretreatment and progression was 234.65 (76.43-387.13) pg/mL and 160.14 (69.47-360.01) pg/mL, respectively, and did not show a significant difference (*P=*0.247).

### Relationship between baseline serum VEGF-A/SII and treatment response of first-line chemotherapy in SCLC patients

**Figure 2:**
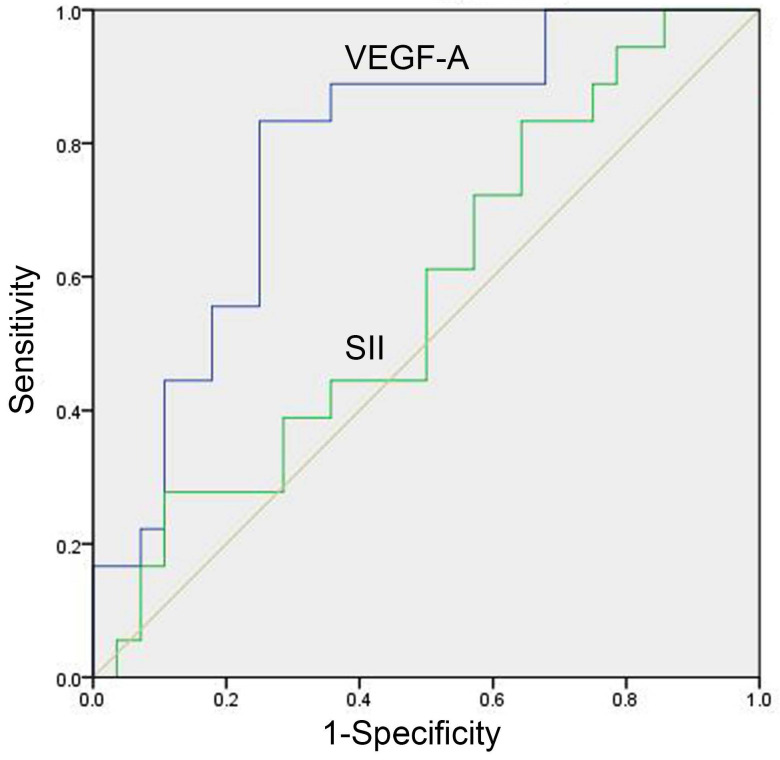
** ROC curve of baseline serum VEGF-A and SII for prediction of treatment response in SCLC patients**. The predictive potentials of baseline VEGF-A and the SII in treatment response were assessed by ROC curves among 28 treatment effective patients (CR+PR) and 18 treatment ineffective patients (SD+PD). The AUC value of VEGF-A was 0.788 (95% CI: 0.642-0.894, *P*<0.001). The SII did not show predictive potential in SCLC (*P*=0.392). Abbreviations: CR: complete response; PD: progressive disease; PR: partial response; SCLC: small cell lung cancer; SD: stable disease; SII: systemic immune-inflammation index; VEGF-A: vascular endothelial growth factor A.

In the study group, there were 46 patients with meaningful efficacy assessment data, of whom 28 were treatment effective (CR+PR) and 18 were treatment ineffective (SD+PD), with baseline VEGF-A of 79.03 (41.34-215.67) and 244.30 (160.13-385.83) pg/mL, respectively, in the two groups, with a significant difference (*P=*0.001, Table 4). The baseline SII of the two groups was 720.40 (488.27-1070.95) (×10^9^/L) and 720.23 (578.12-1671.38) (×10^9^/L), respectively, which was not significantly different (*P=*0.392, Table 4). The AUC of the ROC curve of baseline VEGF-A was 0.788 (95% CI: 0.642-0.894, *P*<0.001, Figure 2). With a cut-off value of 158.71 pg/ml, baseline VEGF-A had a sensitivity of 83.33%, a specificity of 75.00%, a LR+ of 3.33, and a LR- of 0.22. The baseline SII did not show predictive potential in SCLC (*P*=0.392, Figure 2).

### Kaplan-Meier survival analysis

**Figure 3:**
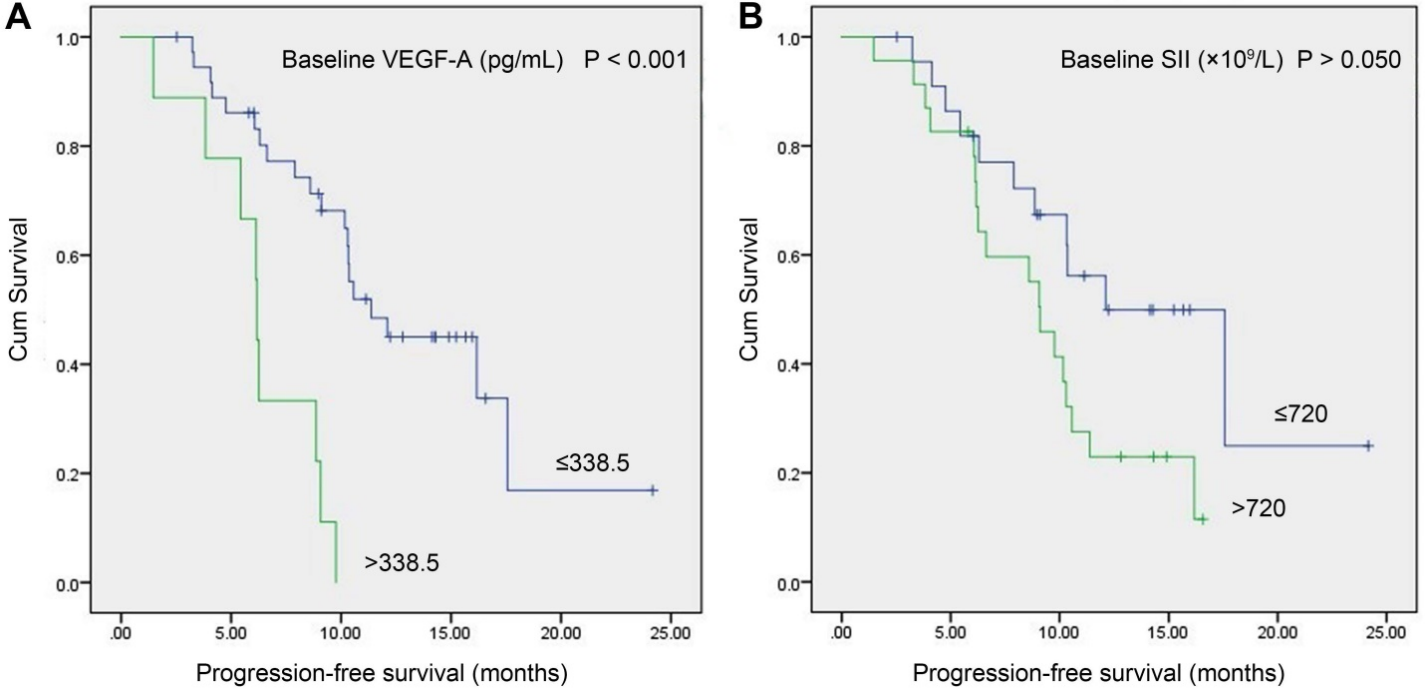
** Kaplan-Meier survival curves of SCLC patients with baseline serum VEGF-A levels vs. baseline SII. (A)** Patients with VEGF-A ≤338.5 pg/mL had significantly longer PFS compared to patients with VEGF-A >338.5 pg/mL (11.37 vs. 6.17 months, *P<*0.001); **(B)** Patients with SII ≤720 (×10^9^/L) had a trend toward longer PFS than patients with SII >720 (×10^9^/L), but the difference did not reach statistical significance (12.10 vs. 9.10 months, *P>*0.050). Abbreviations: SCLC: small cell lung cancer; SII: systemic immune-inflammation index; VEGF-A: vascular endothelial growth factor A.

In the study group, a total of 46 patients with meaningful survival data were obtained. To investigate the relationship between baseline VEGF-A/SII and PFS, we used X-tile software 20 (version 3.6.1, Yale University, New Haven, CT) to determine the optimal cut-off values of 338.5 pg/mL and 720 (×10^9^/L) for VEGF-A and SII, respectively. As shown in Figure 3, patients with VEGF-A ≤338.5 pg/mL had significantly longer PFS compared to those with VEGF-A >338.5 pg/mL (11.37 vs. 6.17 months, *P<*0.001, Figure 3A). Patients with SII ≤720 (×10^9^/L) had a trend toward longer PFS compared to patients with SII >720 (×10^9^/L), but the difference did not reach statistical significance (12.10 vs. 9.10 months, *P>*0.050), Figure 3B).

### Univariate and multivariate Cox regression analyses

As shown in Table 5, the univariate analysis suggested that disease stage (*P=*0.009), treatment response (*P=*0.005), and baseline VEGF-A (*P<*0.001) were significantly associated with PFS in SCLC patients. Variables that were significant in the univariate analysis (*P<*0.05) were included in the multivariate analysis to identify independent prognostic factors. The results indicated that disease stage (HR: 2.438, 95% CI: 1.090-5.452, *P=*0.030), treatment response (HR: 2.209, 95% CI: 1.007-4.845, *P=*0.048), and baseline VEGF-A level (HR: 3.443, 95% CI: 1.330-8.908, *P=*0.011) were independent prognostic factors for PFS in SCLC patients.

## Discussion

SCLC is an aggressive malignancy characterized by rapid growth, early metastatic dissemination and responsiveness to initial therapy. Little progress has been made in the treatment for SCLC over the past few decades; molecularly targeted therapy has failed to yield convincing clinical benefits. Although early studies have shown promising clinical activity of immune checkpoint inhibitors (ICIs), only atezolizumab 21 and durvalumab 22 in combination with chemotherapy improved overall survival (OS) over chemotherapy alone in first-line setting for patients with extensive-stage SCLC in clinical trial up to now. The predictive values of tumor programmed death ligand-1 (PD-L1) expression 23, CD8^+^ tumor-infiltrating lymphocytes (TILs) 23, and high tumor mutational burden (TMB) 24 density have been demonstrated to be associated with ICIs efficacy, however, the immune composition and expression of potentially actionable immunostimulatory targets in SCLC are poorly understood. Cytotoxic chemotherapy remains the cornerstone of SCLC treatment, but there is still a lack of recognized convenient and effective biomarkers to predict the efficacy of chemotherapy and survival of patients with SCLC.

Angiogenesis and immune-inflammation can promote tumor cell proliferation, induce blood vessel formation, inhibit apoptosis, and lead to tumor dissemination 6, 9. Compared with tissue specimens, blood tests not only have the advantages of non-invasive, convenient and repeatable detection, but can also reflect the whole body's angiogenesis and immune-inflammatory status and evaluate the tumor process more comprehensively. In the present study, we first found that serum VEGF-A was significantly higher in SCLC patients than in non-neoplastic patients. It is well known that hypoxia is the major trigger of VEGF-A expression 6, 25. Compared with normal tissues, the growth of tumor tissues requires large amounts of nutrients and oxygen, and the resulting hypoxic TME can activate the PI3K/Akt/HIF-1α pathway, upregulate VEGF-A expression, and stimulate angiogenesis 26. The results of the present study support that a certain degree of hypoxic TME may exist in SCLC patients and can be indirectly reflected by elevated baseline VEGF-A levels. With the high specificity, baseline serum VEGF-A may be able to aid in the diagnosis of SCLC.

We further analyzed the relationship between baseline VEGF-A and the clinicopathological characteristics of SCLC patients and showed that VEGF-A was significantly associated with disease stage and CNS metastasis. In the process of tumor growth, unlimited proliferation requires a constant supply of oxygen. In a hypoxic TME, cancer cells' tolerance is increased, apoptosis is inhibited, and invasion and metastasis are significantly enhanced 27, while enhanced angiogenesis allows cancer cells to spread to distant compartments with the help of neovascularization, contributing to the more rapid progression of SCLC to an advanced stage. More importantly, we found that the median VEGF-A was almost four times higher in patients with CNS metastasis, suggesting that there may be a unique role for angiogenesis in CNS metastasis. Through an *in vitro* study, Argaw et al. 28 found that VEGF-A could degrade the blood-brain barrier by downregulating the tight junction protein Claudin-5, increasing the permeability of brain microvascular endothelial cells (BMECs), while a clinical study by Si et al. 29 found that VEGF-A may be a major factor contributing to blood-brain barrier degradation and CNS metastasis of leukemia cells. To the best of our knowledge, this study is the first clinical study to find a significant association between VEGF-A and CNS metastasis in SCLC patients. Therefore, we believe that serum VEGF-A not only reflects the disease process of SCLC, but high levels of VEGF-A may also be suggestive of CNS metastasis.

To assess the predictive efficacy and prognostic value of baseline VEGF-A, we performed efficacy and survival analyses in patients treated for more than 2 cycles. In the efficacy analysis, we found that after 2 cycles of chemotherapy, patients had significantly lower VEGF-A levels compared with pretreatment levels and returned to pretreatment levels at progression. Baseline VEGF-A levels were significantly higher in treatment-ineffective patients than in treatment-effective patients. Conversely, the Kaplan-Meier survival analysis suggested that patients with low levels of VEGF-A before treatment had significantly longer PFS compared to patients with high levels. The univariate and multivariate analyses confirmed the results of the survival analysis, which concluded that VEGF-A independently predicted poorer PFS in SCLC patients. Treatment resistance and shorter survival are important markers of high tumor malignancy. Zhu et al. 30 found that a hypoxic TME could activate the HIF-1α/VEGF pathway to improve lung cancer cell viability, inhibit apoptosis, and promote angiogenesis and resistance to radiotherapy, leading to a poorer prognosis, while inhibition of this pathway could reverse this process. In the meantime, HIF-1α can induce resistance to chemotherapeutic agents via VEGF-A 31. Increased VEGF level has been demonstrated to be associated with the absence of T cells inside the tumor, and inhibition of VEGF-VEGFR2 interaction can increase T cell infiltration 32. In addition to T cells, tumor-associated macrophages (TAMs) can produce VEGF and matrix metalloproteinase-9 (MMP-9), which promote angiogenesis, tumor invasion and metastasis, leading to a poorer prognosis 33. Thus, serum VEGF-A can reflect the degree of malignancy of SCLC, and high levels of VEGF-A may indicate treatment resistance and shorter survival, which could involve multiple mechanisms.

The prognostic role of VEGF in patients with SCLC has been investigated and shown controversial results in previous studies 13-15, 34, 35. Of these studies, Ustuner et al. 13 and Hasegawa et al. 34 examined the additional predictive value of VEGF and considered that VEGF failed to predict the efficacy of chemotherapy±radiation therapy in patients with SCLC. In comparison to these previous studies in which enzyme-linked immunosorbent assay (ELISA) method was used, we performed the more sensitive and clinical applicable chemiluminescence to detect serum VEGF-A, and successfully revealed the potential of this indicator in predicting the first-line chemotherapeutic response of SCLC. In addition, this study depicts a comprehensive evaluation of the diagnostic, efficacy and prognostic predictive role of VEGF with cut-off value, which may provide a better reference for clinical practice.

The SII, as a novel indicator of immune-inflammation, is considered to have prognostic value in a variety of tumors. For example, Chen et al. 36 retrospectively analyzed 1383 patients with colorectal cancer (CRC) after surgery and found that the SII was an independent prognostic factor for OS and disease-free survival (DFS) of the patients. In addition, similar results have been obtained for the SII in liver 12, esophageal 11, and lung cancers 16, 37. However, in the present study, the SII did not demonstrate an important application in SCLC and was only found to be associated with disease stage and liver metastasis. Lu et al. 38 also rendered the possibility of SII as an independent predictor of postoperative liver metastasis for CRC patients, but the exact association between SII and liver metastasis in patients with advanced cancer is still unclear. Although the population differences and small sample size of the study may affect the assessment of SII, we believe that the SII may have some disadvantages as an indicator of tumor efficacy or prognosis: 1. The SII is susceptible to complications such as bleeding and infection, as well as underlying diseases, which interfere with the judgment of the tumor condition 39, 40. 2. Adverse events after treatment, such as myelosuppression, usually affect the SII, thus reducing its value as a dynamic monitoring indicator. 3. The SII of the patient's peripheral blood may not yet reflect the immune-inflammatory status of the TME. 4. In SCLC, a highly malignant disease, the shorter survival period may make it difficult to adequately observe the impact of the immune-inflammatory status on tumor progression.

In summary, this study found that baseline serum VEGF-A was significantly elevated in primary SCLC patients and significantly correlated with disease stage and CNS metastasis. Baseline VEGF-A was able to predict the treatment response to first-line chemotherapy in SCLC patients and was an independent prognostic factor for SCLC. Baseline SII parameters were significantly correlated with SCLC disease stage and liver metastasis, but are not yet sufficient to be a valid efficacy and prognostic indicator. Given the potential value of VEGF-A as an efficacy/prognostic marker for SCLC and its convenience of clinical testing, it is necessary to expand the sample size, follow patients' overall survival, and collaborate with other centers in the future to confirm this result.

## Figures and Tables

**Table 1:** Relationship between baseline serum VEGF-A/SII and clinicopathological characteristics of SCLC patients

Factors	*N*	VEGF-A (pg/mL)	*P* value	SII (×10^9^/L)	*P* value
**Sex**					
Male	45	103.87 (62.89-251.30)	0.121	720.53 (468.89-952.93)	0.117
Female	14	196.48 (91.80-343.64)		853.08 (660.86-2053.95)	
**Age**					
≤60	22	166.84 (81.03-260.15)	0.594	720.47 (511.97-1033.45)	0.541
>60	37	137.40 (64.50-293.66)		748.30 (556.66-1043.06)	
**Smoking**					
No	35	159.98 (74.67-313.47)	0.405	748.43 (462.94-1100.28)	0.853
Yes	24	117.29 (61.57-256.96)		720.46 (563.22-905.70)	
**ECOG PS**					
0-1	45	144.03 (64.50-243.84)	0.130	782.92 (556.66-1043.06)	0.247
2	14	206.85 (83.28-754.57)		601.34 (322.24-1014.53)	
**Stage**					
II-III	35	96.77 (53.04-192.68)	0.021*	692.50 (462.94-914.05)	0.026*
IV	24	239.53 (78.58-391.01)		851.79 (578.27-1792.05)	
**No. of metastases**					
0-1	49	144.03 (62.89-251.30)	0.151	719.93 (501.57-974.91)	0.090
≥2	10	215.32 (73.61-694.19)		919.17 (700.55-1991.50)	
**CNS metastasis**					
No	55	137.40 (67.23-239.99)	0.012*	748.30 (556.42-982.95)	0.976
Yes	4	531.31 (252.11-800.00)		920.52 (314.41-2728.36)	
**Liver metastasis**					
No	51	144.03 (65.51-262.61)	0.250	719.93 (474.83-966.88)	0.018*
Yes	8	215.32 (78.58-402.59)		1245.83 (764.12-3424.75)	

Abbreviations: CNS: central nervous system; ECOG PS: eastern cooperative oncology group performance status; SCLC: small cell lung cancer; SII: systemic immune-inflammation index; VEGF-A: vascular endothelial growth factor A; * *P<*0.05.

**Table 2:** Dynamic changes of serum VEGF-A in SCLC patients before and after treatment

*N*	VEGF-A (pg/mL)			χ^2^	*P* value
	Pretreatment	1 cycle after	2 cycles after		
46	148.06(58.46-299.45)	90.59 (42.92-218.58)	90.14 (49.94-132.61)	6.110	0.047*

Abbreviations: SCLC: small cell lung cancer; VEGF-A: vascular endothelial growth factor A; * *P<*0.05.

**Table 3:** Dynamic changes of serum VEGF-A in patients with SCLC before and at progression of treatment

N	VEGF-A (pg/mL)		Z	*P* value
	Pretreatment	Progression		
29	234.65 (76.43-387.13)	160.14 (69.47-360.01)	-1.157	0.247

Abbreviations: SCLC: small cell lung cancer; VEGF-A: vascular endothelial growth factor A.

**Table 4:** Comparison of baseline serum VEGF-A and SII in SCLC patients with different treatment response

Response	*N*	VEGF-A (pg/mL)	Z	*P* value	SII (×10^9^/L)	Z	*P* value
CR+PR	28	79.03 (41.34-215.67)	-3.264	0.001**	720.40 (488.27-1070.95)	-0.855	0.392
SD+PD	18	244.30 (160.13-385.83)			720.23 (578.12-1671.38)		

Abbreviations: CR: complete response; PD: progressive disease; PR: partial response; SCLC: small cell lung cancer; SD: stable disease; SII: systemic immune-inflammation index; VEGF-A: vascular endothelial growth factor A; **P<0.01.

**Table 5:** Univariate and multivariate Cox regression analyses of PFS in SCLC patients

Variables	Univariate			Multivariate		
	HR	95% CI	*P* value	HR	95% CI	*P* value
**Sex**						
Male vs. Female	1.121	0.486-2.585	0.788	-	-	-
**Age**						
>60 vs. ≤60	0.727	0.349-1.518	0.396	-	-	-
**Smoking**						
Yes vs. No	0.653	0.304-1.403	0.275	-	-	-
**ECOG PS**						
2 vs. 0-1	1.331	0.582-3.045	0.498	-	-	-
**Stage**						
IV vs. II-III	2.849	1.297-6.261	0.009**	2.438	1.090-5.452	0.030*
**No. of metastases**						
≥2 vs. 0-1	2.259	0.896-5.697	0.084	-	-	-
**CNS metastasis**						
Yes vs. No	0.251	0.057-1.102	0.067	-	-	-
**liver Metastasis**						
Yes vs. No	0.581	0.217-1.557	0.280	-	-	-
**Treatment response**						
SD+PD vs. CR+PR	2.933	1.390-6.190	0.005**	2.209	1.007-4.845	0.048*
**Baseline VEGF-A (pg/mL)**						
>338.5 vs. ≤338.5	5.397	2.183-13.340	<0.001**	3.443	1.330-8.908	0.011*
**Baseline SII (×10^9^/L)**						
>720 vs. ≤720	2.134	0.982-4.636	0.056	-	-	-

Abbreviations: CI: confidence interval; CNS: central nervous system; CR: complete response; ECOG PS: eastern cooperative oncology group performance status; HR: hazard ratio; PD: progressive disease; PFS: progression-free survival; PR: partial response; SCLC: small cell lung cancer; SD: stable disease; SII: systemic immune-inflammation index; VEGF-A: vascular endothelial growth factor A; *P<0.05; **P<0.01.

## Abbreviations

AUC: areas under the curve
BMEC: brain microvascular endothelial cell
CI: confidence interval
CNS: central nervous system
CR: complete response
CRC: colorectal cancer
DFS: disease-free survival
ECOG PS: eastern cooperative oncology group performance status
ELISA: enzyme-linked immunosorbent assay
ICIs: immune checkpoint inhibitors
HR: hazard ratio
LR+: positive likelihood ratio
LR-: negative likelihood ratio
MMP-9: matrix metalloproteinase-9
OS: overall survival
PD: progressive disease
PD-L1: programmed death ligand-1
PFS: progression-free survival
PR: partial response
RECIST: response evaluation criteria in solid tumors
ROC: receiver operating characteristic curve
SCLC: small cell lung cancer
SD: stable disease
SII: systemic immune-inflammation index
TAMs: tumor-associated macrophages
TILs: tumor-infiltrating lymphocytes
TMB: tumor mutational burden
TME: tumor microenvironment
VEGF-A: vascular endothelial growth factor A
